# Circular RNA hsa-circ-0016347 promotes proliferation, invasion and metastasis of osteosarcoma cells

**DOI:** 10.18632/oncotarget.16104

**Published:** 2017-03-10

**Authors:** Hao Jin, Xin Jin, Hong Zhang, Wenbo Wang

**Affiliations:** ^1^ Department of Orthopaedics, The First Affiliated Hospital of Harbin Medical University, Harbin, Heilongjiang Province, People's Republic of China; ^2^ Department of Ophthalmology, The First Affiliated Hospital of Harbin Medical University, Harbin, Heilongjiang Province, People's Republic of China

**Keywords:** circ-0016347, mir-214, caspase-1, osteosarcoma, therapy

## Abstract

Circular RNAs (circRNAs) are a novel class of non-coding RNA which have recently shown huge capabilities in the regulation of gene expression at the post-transcriptional level. Growing evidence has indicated that circRNAs could serve as competing endogenous RNAs (ceRNAs) to bind with microRNAs (miRNAs) and to inhibit the activity and function of the targeted miRNAs. Here, we demonstrated that circ-0016347 acted as a positive regulator in osteosarcoma cells proliferation and invasion. Moreover, circ-0016347 was identified as a sponge of miR-214 that upregulated the expression of caspase-1, which is the functional target of miR-214. Our study provides novel insight into the regulatory mechanism of circ-0016347 and its downstream targets in proliferation, invasion and metastasis of osteosarcoma cells, which will facilitate further development in the therapy of osteosarcoma.

## INTRODUCTION

Osteosarcoma is one of the most devastating malignant neoplasms that arises from primitive transformed cells of mesenchymal origin in children and adolescents [1]. Although the worldwide incidence of osteosarcoma is approximately 3 to 4 new cases per million [2], osteosarcoma is highly aggressive and rapidly metastasizes, and the efficacy of conventional therapy is seriously limited, which results in poor overall survival in patients. Trials with the addition of biological therapies such as interferon, mifamurtide and others have been carried out with the aim of improving survival. A phase-3 randomized study has shown that the addition of mifamurtide has increased the survival to 78 % in nonmetastatic patients. However, the survival in metastatic patients is still dismal. and the molecular mechanisms of this disease are poorly understood. Some new insights in the understanding of the molecular mechanisms for the development of and metastatic potential of osteosarcoma, may be promising for treatment. Understanding the mechanisms of osteosarcoma and finding new molecular markers may help prevent osteosarcoma formation, and reduce risks of complications [3–5].

CircRNAs are a class of endogenous RNAs, which are characterized by covalently closed loop structures without polarity or a polyadenylated tail [6, 7]. CircRNAs were identified many years ago, but were generally considered to be errors in splicing [8]. In recent years, with the development of high-throughput sequencing and novel computational approaches, circRNAs have been identified as having important roles in the regulation of gene expression at the post-transcriptional level [9]. Accordingly, it has been demonstrated that many circRNAs serve as ceRNAs to inhibit the activity of miRNAs [10]. Since miRNAs have been largely reported to regulate gene expression, investigating the function of circRNAs will enable us to better understand the mechanisms underlying the occurrence and development of the associated diseases. Based on the findings in previous studies that potassium voltage-gated channel subfamily H member 1 (KCNH1) was overexpressed in osteosarcoma and promoted the proliferation and invasion of osteosarcoma [11–13] and on our profile of the miRanda, PITA, RNAhybrid databases to explore the corresponding circRNAs of KCNH1, we speculated that hsa-circ-0016347 may be a potential regulator of osteosarcoma progression.

In comparison to circRNAs, miRNAs have been extremely well studied. There is abundant evidence that miRNAs are involved in the occurrence and development of tumors, thereby being potential cancer biomarker candidates [14]. A large variety of miRNAs have been demonstrated to be closely associated with osteosarcoma [15–17]. In previous studies, miR-214 was identified as a tumor promoter in osteosarcoma [18, 19]. However, the function and regulatory mechanisms of miR-214 in the progression of osteosarcoma have not been satisfactorily explained and require further investigated.

It has been widely accepted that there is a strong association between inflammation and cancer. In the tumor microenvironment, inflammatory cytokines influence almost every aspect of tumor progression including invasion and metastasis abilities [20]. Caspase-1, a kind of cysteine proteases, has been shown to proteolytically cleave and activate inflammatory cytokines such as IL-1β and IL-18, which subsequently led to the formation of an inflammatory microenvironment. Our previous experiments have found that the expression of caspase-1 was higher in osteosarcoma tissues than that in the matched adjacent non-tumor tissues. However, to the best of our knowledge, the role of caspase-1 and its upstream regulators in osteosarcoma have not been clarified. Therefore, revealing the involvement of circ-0016347/miR-214/caspase-1 in the inflammation-related mechanisms in the progression of osteosarcoma is vitally important for the effective treatment of osteosarcoma.

## RESULTS

### circ-0016347 levels are elevated in osteosarcoma

**Figure 1:**
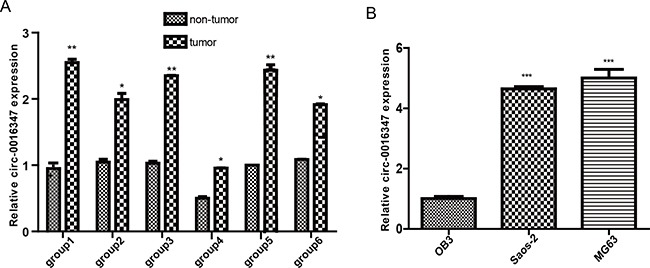
The expression levels of circ-0016347 are elevated in osteosarcoma **(A)** Circ-0016347 expression levels in six groups of osteosarcoma tissues are significantly higher than that in matched non-tumor tissues as determined by real-time PCR. **(B)** Circ-0016347 mRNA expression levels in OB3, MG63 and Saos-2 osteosarcoma cells. Data are expressed as the mean ± SEM. n=3. * p < 0.05, ** p <0.01, *** p < 0.001 when compared to the control group.

We randomly collected osteosarcoma tissues and adjacent non-tumor tissues from 6 patients. The circ-0016347 expression levels were measured by qRT-PCR. The results showed that circ-0016347 expression levels were higher in osteosarcoma tissues than in the adjacent non-tumor tissues (Figure 1A). Moreover, we found that the expression levels of circ-0016347 were obviously elevated in osteosarcoma cell lines (Saos-2, MG-63) compared to a normal osteoblast OB3 cell line (P < 0.05) (Figure 1B). These findings imply that circ-0016347 levels have a strong positive correlation with the presence of osteosarcoma.

### circ-0016347 promotes the proliferation, invasion and metastasis of osteosarcoma cells

**Figure 2:**
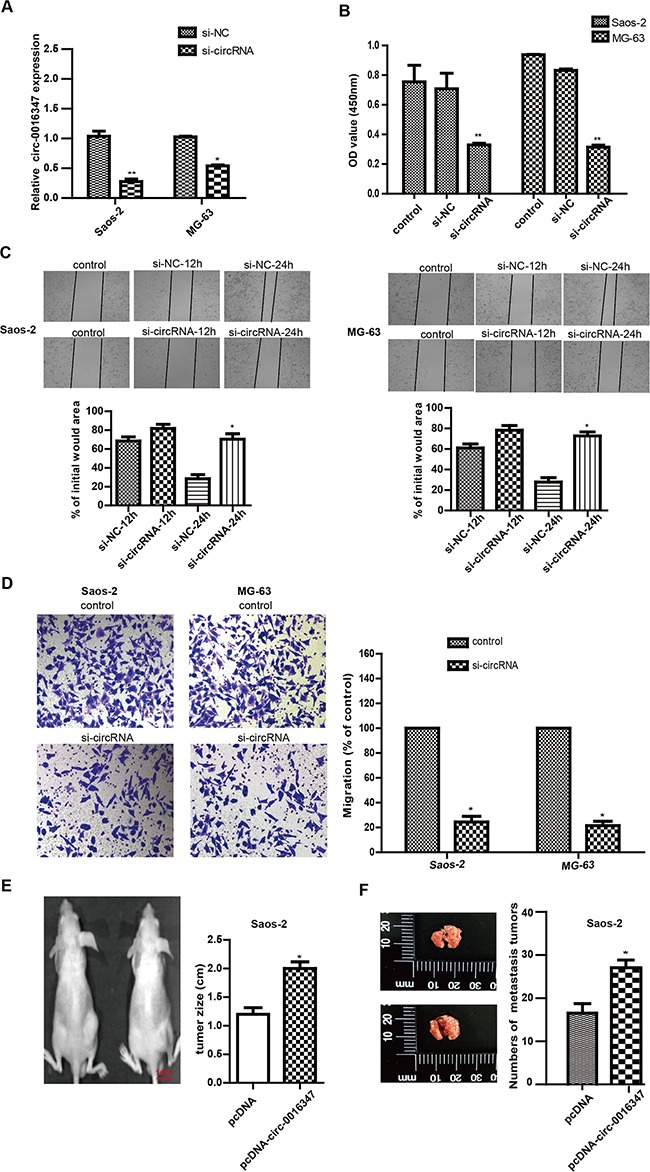
Circ-0016347 knockdown inhibits the proliferation, invasion and metastasis of osteosarcoma cells **(A)** The expression levels of circ-0016347 in Saos-2 and MG-63 osteosarcoma cells after transfection with circ-0016347 siRNAs. **(B)** Cell proliferation determined by CCK-8 assay. **(C-D)** Knockdown of circ-0016347 also reduced cell proliferation and invasion determined by wound healing **(C)** and transwell assays **(D)**. **(E-F)** The Saos-2 cells were transfected with circ-0016347 overexpression plasmid (pcDNA-circ-0016347) (left in the figure2E) or empty vector plasmid (pcDNA) (right in the figure2E) and then planted under the skin of mice or injected into the tail vein of mice. The tumor sizes in the xenograft mouse model **(E)**, and the numbers of pulmonary metastasis tumors **(F)**. Data are expressed as the mean± SEM. n=3. * p < 0.05, ** p < 0.01 when compared to the control group.

To determine the functional role of circ-0016347 in the proliferation, invasion and metastasis of osteosarcoma cells, circ-0016347 siRNAs were used to knockdown circ-0016347 in MG-63 and Saos-2 cells, which have high endogenous circ-0016347 expression. Then, we assessed the effect of circ-0016347 on the proliferation and invasion of osteosarcoma cells. qRT-PCR revealed that circ-0016347 were significantly downregulated by circ-0016347 siRNA transfection compared with the negative siRNA control (Figure 2A). CCK-8 results showed that circ-0016347 knockdown dramatically reduced MG-63 and Saos-2 cell proliferation compared with control cells (Figure 2B). Knockdown of circ-0016347 also reduced the cell proliferation (Figure 2C) and invasion (Figure 2D) in MG-63 and Saos-2 cells compared to cells transfected with negative control siRNA. Furthermore, in order to see the function of circ-0016347 *in vitro*, we transfected pcDNA-circ-0016347 or pcDNA into the Saos-2 cells and then injected subcutaneously into the back of mice or the tail vein of mice. The results showed that the tumor sizes (E) or the numbers of pulmonary metastasis tumors (F) were distinctly increased in the circ-0016347 overexprssion mice than that in the control mices. Together these data indicated that circ-0016347 played a positive role in the proliferation, invasion and metastasis of osteosarcoma cells.

### circ-0016347 promotes caspase-1 expression and cell proliferation in osteosarcoma

**Figure 3:**
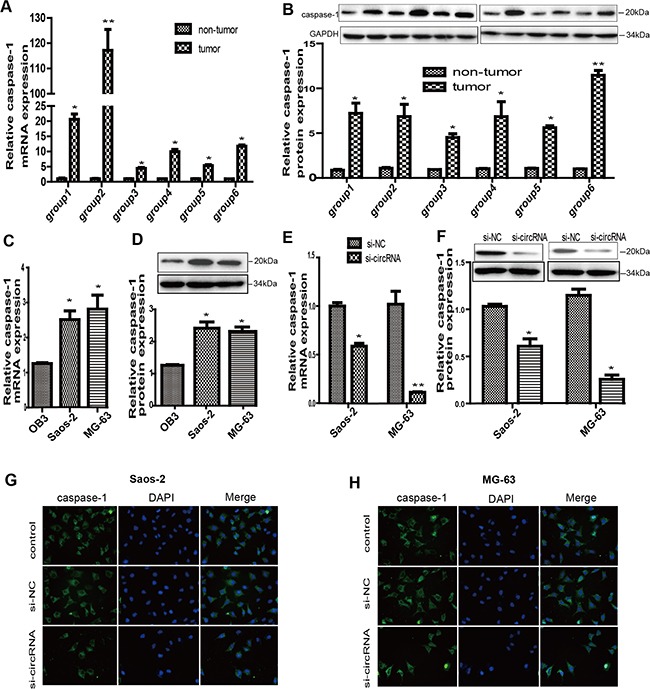
Circ-0016347 was positively related to the expression of caspase-1 in osteosarcoma **(A-B)** Caspase-1 expression levels are dramatically increased in osteosarcoma tissues groups compared to that in the non-tumor groups. **(C-D)** The mRNA and protein expression levels of caspase-1 are elevated in both Saos-2 and MG-63 osteosarcoma cells compared to OB3 osteoblast cells. **(E-F)** The mRNA and protein expression levels of caspase-1 are obviously decreased in MG-63 and Saos-2 cells after transfection with circ-0016347 siRNAs. **(G-H)** Immunocytochemistry showed reduced caspase-1 expression by inhibition of circ-0016347. Data are expressed as the mean ± SEM. n=3. * p < 0.05, ** p< 0.01 when compared to the control group.

A large body of evidence has documented that caspase-1 is important for the formation of the tumor microenvironment,contributing to the cell proliferation and invasion of osteosarcoma. Therefore, we attempted to analyze the relationship between circ-0016347 and caspase-1 in osteosarcoma. The previous results have shown that circ-0016347 levels were obviously elevated in six osteosarcoma tissues compared to matched adjacent non-tumor tissues (Figure 1A). Consistent with our hypothesis, the expression levels of caspase-1 were notably increased in the high circ-0016347 osteosarcoma tissue group compared to the levels in the low circ-0016347 group (Figure 3A-3B). Accordantly, caspase-1 levels were higher in MG-63 and Saos-2 cells than in OB3 cells (Figure 3C-3D). Furthermore, we silenced circ-0016347 and then evaluated caspase-1 expression levels in MG-63 and Saos-2 cells. We found that the knockdown of circ-0016347 by siRNA resulted in reduced caspase-1 expression compared to cells transfected with control siRNAs (Figure 3E-3F). We also performed immunocytochemistry assays to further confirm the positive correlation between the presence of circ-0016347 and proliferation of osteosarcoma cells; Figure 3G-3H reveal that the proliferation ability of osteosarcoma cells was weakened by the knockdown of circ-0016347. Taken together, these results illustrated that circ-0016347 probably contributes to the proliferation and invasion of osteosarcoma by allowing an increased expression of caspase-1 with oncogenic potential.

### Caspase-1 is a downstream target of miR-214

**Figure 4:**
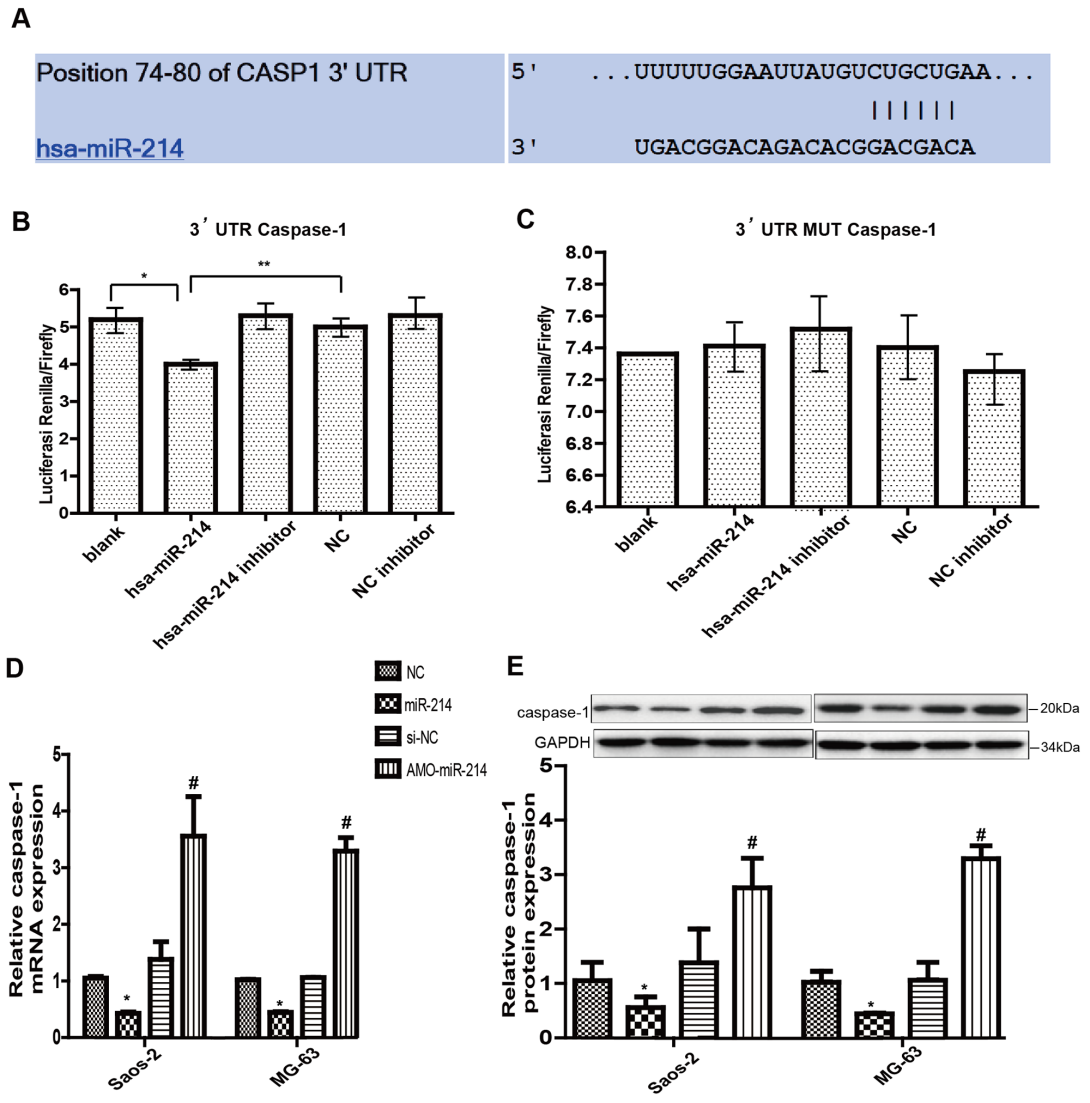
Caspase-1 is a downstream target of circ-0016347 **(A)** The bioinformatic prediction (TargetScan Human 5.1) of the putative binding sites of caspase-1 3′-UTR sequence with miR-214. **(B-C)** Effects of blank, hsa-miR-214, hsa-miR-214 inhibitor, NC (negative control) and NC inhibitor on the luciferase activity of caspase-1 3′-UTR sequence or mutant 3′-UTR sequence by a luciferase reporter assay. **(D-E)** The mRNA and protein levels of caspase-1 decrease when Saos-2 and MG-63 osteosarcoma cells are transfected with miR-214 and increase when the cells are transfected with the miR-214 inhibitor (AMO). Data are expressed as the mean ± SEM. n=3. * p < 0.05, ** p < 0.01,when compared to the control group.

MiRNAs, which regulate the stability and translational efficiency of partially complementary target mRNAs, are frequently aberrantly expressed in tumors, eventually promote proliferation in cell lines, and accelerate angiogenesis and tumorigenesis [14, 21]. In recent years, there has been an increasing interest in the functional crosstalk between circRNAs and miRNAs. Therefore, in this study, we tried to find a certain miRNA, which has functional relationship with circ-0016347 and is involved in the regulation of caspase-1 expression. To identify the potential miRNA that targets caspase-1, we used a bioinformatics tool (TargetScan Human 5.1) to examine potential complementary base pairings between caspase-1 and miRNAs. The bioinformatics prediction revealed that the caspase-1 3, UTR sequence has putative miR-214 binding sites (Figure 4A). Indeed, reduction of luciferase activity was observed upon the combination of miR-214 and caspase-1 3, UTR (p< 0.01, Figure 4B). Additionally, it has been previously reported that miR-214 could promote osteosarcoma tumor growth and metastasis [19, 22, 23]. We thus focused on miR-214 in our study. To detect the effect of miR-214 on the expression of caspase-1, we performed gain-of-function and loss-of-function assays. We found that the expression levels of caspase-1 and the associated mRNA were dramatically decreased after cells were transfected with miR-214 mimic. In contrast, miR-214 inhibitor significantly increased the level of caspase-1(Figure 4C-4D). These results confirm that caspase-1 is the direct target of miR-214.

### The functional crosstalk between circ-0016347 and miR-214

**Figure 5:**
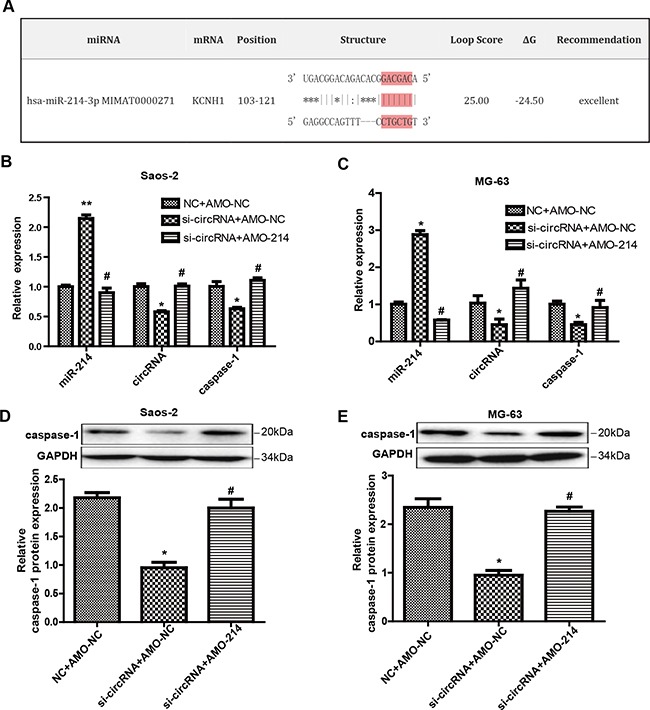
Circ-0016347 increases the level of caspase-1 through miR-214 **(A)** The putative nucleotide-binding sites between KCNH1 (the corresponding linear sequence of circ-0016347) and miR-214-3p fromthe bioinformatics tool miRanda. **(B-C)** The expression level of miR-214 was visibly elevated in Saos-2 and MG-63 cells after being transfected with circ-0016347-siRNA. The caspase-1 mRNA expression levels are reduced by the inhibition of circ-0016347 and reversed by the miR-214 inhibitor (AMO-214). **(D-E)** The protein expression levels of caspase-1 are in accordance with its mRNA expression levels. Data are expressed as the mean ± SEM. n=3. * p < 0.05, ** p< 0.01 when compared to the control group, # p < 0.05 vs. si-circRNA groups.

Increasing evidence has have shown that circRNAs sequester miRNAs to terminate regulation of their target genes [24]. Thus, we speculated that circ-0016347 could target certain miRNA to inhibit their expression or activity. To screen for miRNAs that could be combined with circ-0016347, we profiled the public databases miRanda, PITA, and RNAhybrid and found that miR-214 had binding sites with human potassium voltage-gated channel subfamily H member 1 (KCNH1), which was the corresponding linear sequence of circ-0016347 (Figure 5A). To further examine the relationships between circ-0016347 and miR-214 and between miR-214 and its target caspase-1, MG-63 and Saos-2 cells were transfected with si-circRNA or co-transfected with si-circRNA and miR-214 inhibitor. Then, we detected the expressions of circ-0016347, miR-214 and caspase-1 by qRT-PCR or western blot. The results show that the levels of miR-214 were significantly elevated in the cells transfected with si-circRNA compared with negative control cells, however the miR-214 inhibitor (AMO-214) significantly reversed the high levels of miR-214 caused by si-circ-0016347 (Figure 5B-5C). The caspase-1 protein expression levels are in accordance with its mRNA expression levels (Figure 5D-5E).

Circ-0016347 acts as a natural miR-214 sponge, and miR-214 has similar suppressive effects on the expression of circ-0016347. In Figure 5B-5C, it can be seen that the circ-0016347 expression level was partly increased when miR-214 was inhibited compared to cells merely transfected with si-circRNA. Furthermore, the evidence also showed that circ-0016347 is positively correlated with caspase-1, which could be reversed by miR-214 inhibitor. Collectively, these data illustrate that circ-0016347 negatively correlates with miR-214 and indirectly influences the expression of caspase-1 in the osteosarcoma cells.

## DISCUSSION

Osteosarcoma is the most common type of primary bone malignancy. Because of the lack of early diagnosis and effective therapeutic approaches, the mortality rates remain worryingly high for several years. Novel in-depth studies are essential to diagnose the osteosarcoma in earlier stages, which is the key to fighting against the disease.

CircRNAs represent a novel class of regulatory non-coding RNA that have only recently been identified and cataloged. Emerging evidence has indicated that circRNAs are widely expressed in biological systems and are characterized by highly conserved sequences and a high degree of stability in mammalian cells [25]. These properties provide circRNAs with the potential to become perfect biomarkers in the diagnosis of various cancers [26]. However, as a newly appreciated form of non-coding RNA with great potential implications in clinical and research fields, there are only a few studies on the relationship between circRNAs and tumors. Therefore, exploration of the function relationship between circular RNA and cancer is urgently needed and valuable. In this study, we found that the level of circ-0016347 was significantly higher in osteosarcoma cells compared to normal groups, for both *in vitro* and *in vivo*. However, the specific function of circ-0016347 in the occurrence and development of osteosarcoma remains largely elusive.

There have been a number of studies on the complicated relationship between inflammation and cancer. It is well-known that chronic inflammation could promote the occurrence and development of tumors [27, 28]. Additionally, inflammation mediates systemic immunosuppressionthat is a major obstacle for effective treatment of cancers, including osteosarcoma [29, 30]. Therefore, clarifying the key mechanism involved in the regulation of inflammatory factors will be beneficial for the understanding and better treatment of osteosarcoma. Among the multiple inflammatory factors and related proteases, we focused on the expression of caspase-1 on account of its essential role in the formation of tumor inflammatory microenvironments. Caspase-1 cleaves and activates the proinflammatory cytokines IL-1β and IL-18 into their mature peptides, which contribute to the down-stream inflammatory response and formation of tumor microenviroment [31, 32]. Interestingly, in our previous study, we found that caspase-1 was significantly elevated in osteosarcoma patients. Although it is widely accepted that caspase-1 has an anti-cancer effect, there are some other researches demonstrated that caspase-1 also has the potential to promote tumor invasiveness and metastases [33].

MicroRNAs play important roles in the regulation of various biological processes, including cell proliferation, apoptosis, metastasis and inflammation [34, 35]. Specifically, many studies have indicated that various miRNAs, such as miR-143, miR-214, and miR-21, were related to the development of osteosarcoma [22, 23, 36, 37]. Interestingly, according to the prediction results of a bioinformatics tool (TargetScan Human 5.1), we found potential complementary base pairing between miR-214 and caspase-1 3′UTR. Luciferase activity assays further validated the targeted relationship between miR-214 and caspase-1.

Emerging researches show that there is functional crosstalk between circRNAs and miRNAs, but the effects of the interaction of circRNAs with miRNAs on the progression of osteosarcoma remain unknown. According to the prediction results of the bioinformatics software and previous results, we found that miR-214 could interact with both circ-0016347 and caspase-1. In addition, the expression level of miR-214 was obviously increased after circ-0016347 was inhibited. This suggests that circ-0016347 competitively binds to miR-214 and inhibitsmiR-214 activity, resulting in increased expression levels of the targeted gene of caspase-1. These results implied that circ-0016347 promoted osteosarcoma cell proliferation, invasion and metastasis, at least partly by influencing the formation of the inflammatory microenvironment through the miR-214/caspase-1 axis.

In summary, our study demonstrates that the functional crosstalk between circ-0016347 and miR-214, as well as the down-stream target caspase-1, are critically involved in the proliferation, invasion and metastasis of osteosarcoma cells. Circ-0016347 acts as a miRNA sponge to directly inhibit the activity and function of miR-214 and then subsequently increases the expression of the down-stream target caspase-1 in osteosarcoma cells. This report revealed a novel mechanism of hsa-circ-0016347 and miR-214 in osteosarcoma. It might contribute to establishing potential therapeutic strategies for osteosarcoma.

## MATERIALS AND METHODS

### Tissue samples

Six pairs of tissue samples were collected from patients diagnosed with osteosarcoma who underwent surgery at The First Affiliated Hospital of Harbin Medical University, China. The samples were snap-frozen and stored at −80°C until total RNA or protein extraction. All patients provided signed consent to the research. The Research Ethics Committee at Harbin Medical University approved the study.

### Cell culture and transfection

Human osteosarcoma cell lines Saos-2 and MG-63 and the human osteoblast cell line hFOB (OB3) were purchased from the Chinese Cell Bank of the Chinese Academy of Sciences (Shanghai, China). Cells were cultured in Dulbecco's Modified Eagle Medium (DMEM; HyClone, USA) supplemented with 10% (v/v) fetal bovine serum (FBS; Gibco, USA) in an atmosphere of 95% humidified air and 5% CO_2_ at 37°C. Cells were investigated within 8 h of harvest. Si-circRNAs against human circ-0016347 were constructed by RIBOBIO (Guangzhou, China). Knockdown and overexpression of circ-0016347 and miR-214 were obtained from Invitrogen (Carlsbad, CA, USA). All cell transfections were performed according to the manufacturer's protocol (X-tremeGENE siRNA Transfection Reagent, Roche, USA).

### Cell proliferation assay

Cell proliferation was determined by the cell count kit-8 (CCK-8) cell proliferation kits according to the manufacturer's instructions. MG-63 and Saos-2 cells were seeded in 96-well plates at 1 × 10^4^ cells/well and maintained for 24 h. CCK-8 solution (10 μl) was added to each well and cells were incubated at 37°C for 2 h. The absorbance at 450 nm was evaluated using a microplate reader. The data are representative of three individual experiments carried out in triplicate.

### Wound healing assays

Osteosarcoma cells were seeded into six-well plates and grown to 80–90% confluence. A wound was produced by a straight scratch with a 200-μL sterile pipette tip. The osteosarcoma cells were then rinsed with phosphate-buffered saline (PBS) to remove the floating cells. Images were captured within 12h, 24 h post-wound. The relative distance of cell migration to the scratched area was measured and a healing percentage was calculated. Each test was carried out in triplicate for more than two independent experiments.

### Transwell assays

Transwell chambers containing polycarbonate membrane filters with a 24-well 8-μm pore size (Corning Company, USA) were coated with Matrigel. In each well, 40 μl of Matrigel was added to an insert and dried in a 37°C incubator for 30 minutes to form a thin gel layer. Then, 24 h after MG63 and Saos-2 cells transfected with si-circRNA, the cells were resuspended with reduced serum DMEM/F12 and were adjusted to 2.5 × 10^5^ cells/mL. After 24 h, Matrigel and cells remaining on the upper side of the membrane were wiped off, and the cells that had migrated to the bottom surface of the membrane were fixed in 4% paraformaldehyde in PBS. Once fixed, the cells were stained with crystal violet for 10 min at room temperature. Four randomly selected fields were captured using a fluorescence microscope (Nikon company, Japan) to calculate the number of cells that had successfully invaded and transmigrated the Matrigel. Data were expressed as the average number of cells per insert.

### Xenograft mouse model and pulmonary metastasis model establishment

The experimental were approved by the Ethic Committee of Harbin Medical University, China. Animal care and all animal related experimental procedures were conducted in accordance with the guidelines of the INHA Institutional Animal Care and Use Committee (INHA IACUC). Male BALB/c mice, 5 to 6 weeks old weighing 18-20 g were used. Firstly, Saos-2 cells were transfected with circ-0016347 overexpression plasmid (pcDNA-circ-0016347) or empty vector plasmid (purchased from Geneseed Biotech Co.,Ltd). After 48h, the Saos-2 cells were suspended in 100μl serum-free DMEM and then injected subcutaneously into the back of mice or injected into the tail vein of mice. After 2 weeks, the tumor were isolated and the longest diameters or the numbers of pulmonary metastasis tumors were calculated.

### RNA extraction and quantitative PCR

Total RNA was extracted by Trizol reagent (Invitrogen). PCR primers were purchased from Invitrogen. The concentration of RNA was confirmed by a NanoDrop Spectrophotometer (NanoDrop Technologies, Wilmington, DE). For miR-214, cDNA was synthesized from 5 ng of total RNA by a TaqMan miRNA reverse transcription kit (Applied Biosystems, USA) to determine the miR-214 levels. The results were normalized to U6 levels and calculated by using the 2^−ΔΔCT^ method. For circ-0016347 and caspase-1,cDNA was synthesized using a reverse transcriptase kit (Applied Biosystems, USA). Real-time PCR was performed using the SYBR Green PCR Master Mix (ToYoBo, Japan) and the ABI 7500 Sequence Detection System (Life Technologies, USA). PCR amplification was performed in a 20 μl reaction volume including 2 μl cDNA, 6 μl DEPC, 10μl SYBR Green Master Mix, 1 μl forward primer and 1 μl reverse primer. PCR conditions were as follows: 40 cycles of 95°C for 15 s, 60°C for 15 s, and 72°C for 45 s. Actin served as an internal control for circ-0016347 and caspase-1, and the results were calculated by using the 2^−ΔΔCT^ method. The primers sequences are listed in Table 1.

**Table 1:** The primers used for qRT-PCR

Names	Sequence (5′-3′)	Length (bp)
GAPDH	AAGAAGGTGGTGAAGCAGGC	20
	TCCACCACCCTGTTGCTGTA	20
CASP1	ACACGTCTTGCCCTCATTATCT	22
	ATAACCTTGGGCTTGTCTTTCA	22
U6	CTCGCTTCGGCAGCACATATACT	23
	ACGCTTCACGAATTTGCGTGTC	22
miR-214	TATACATCAAACAGCAGGCACA	22
	CATTCGATCTTCTCCACAGTCTC	23

### Luciferase assay

The luciferase reporter plasmid or empty vector was transfected into cells together with the encoding gene plasmid. Luciferase activities were measured at 48-h post-transfection using the Dual-Luciferase Reporter Assay System (Promega, Madison, WI) according to the manufacturer's protocol. Renilla luciferase activity was normalized against firefly luciferase activity and presented as percent inhibition. All transfection experiments were performed in triplicate and repeated at least 3 times.

### Western blotting analysis

Western blotting assays were used to detect the expression levels of caspase-1 proteins. The total protein of the tissues and cells was harvested with radioimmunoprecipitation assay buffer (RIPA) containing 1% protease inhibitor (Sigma, USA). For each sample, protein (100 μg) was separated using 12% SDS–PAGE and then transferred into nitrocellulose membranes (Poll,USA). Afterwards, the membrane was blocked with 5% nonfat milk (BD Biosciences) and 0.1% Tween 20 in tris-buffered saline and immunoblotted overnight using caspase-1 primary antibodies at 4°C with gentle shaking. After that, a fluorochrome labelled secondary antibody (Alexa Fluor 800, LI-COR, USA) was used to identify the caspase-1 antibody. Immunoreactivity was detected with the Odyssey fluorescent scanning system (LI-COR) and analyzed by Image Studio software.

### Immunocytochemistry

To investigate the effect of circ-0016347 on the expression of caspase-1, cells were transfected with si-circ-0016347 or nonsense siRNA. After 24h, cells were fixed with 4% paraformaldehyde for 10 min at room temperature and washed with PBS before permeabilization by 0.5% Triton X-100 for 10 min. Nonspecific binding sites were blocked with 5% BSA containing 0.5% Trion X for 1 hour prior to incubation with caspase-1 antibodies overnight at 4°C. The cells were washed three times with PBS and incubated with secondary antibodies for 1 hour at room temperature. The nuclei were visualized by DAPI counterstaining for 5 min. Immunofluorescence images were observed utilizing an inverted fluorescencemicroscope (Olympus, Japan).

### Statistical analysis

Data were expressed as the mean ± standard error of mean (mean ± SEM) and analyzed with SPSS 13.0 software. Statistical comparisons between two groups were performed using Student's t-test. Statistical comparisons among multiple groups were performed using analysis of variance (ANOVA). A two-tailed P < 0.05 was taken to indicate a statistically significant difference.
